# Novel Biochemical Markers of Neurovascular Complications in Type 1 Diabetes Patients

**DOI:** 10.3390/jcm9010198

**Published:** 2020-01-10

**Authors:** Bogusz Falkowski, Anita Rogowicz-Frontczak, Ewelina Szczepanek-Parulska, Aleksandra Krygier, Elzbieta Wrotkowska, Aleksandra Uruska, Aleksandra Araszkiewicz, Marek Ruchala, Dorota Zozulinska-Ziolkiewicz

**Affiliations:** 1 Department of Internal Medicine and Diabetology, Mickiewicza 2, Poznan University of Medical Sciences, 60-834 Poznan, Poland; 2 Department of Endocrinology, Metabolism and Internal Medicine, Przybyszewskiego 49, Poznan University of Medical Sciences, 60-355 Poznan, Poland

**Keywords:** type 1 diabetes mellitus, neurovascular, microvascular, epidermal growth factor, growth/differentiation factor 15, interleukin 29, EGF, GDF-15

## Abstract

Type 1 diabetes mellitus (T1DM) is associated with chronic complications, which are the result of neurovascular changes. There is still a lack of universal biochemical markers of microvascular damage. The present study aimed to investigate whether selected inflammatory proteins are related to the prevalence of microvascular complications in adult T1DM patients. The following markers were determined in a group of 100 T1DM participants: epidermal growth factor (EGF), metalloproteinase 2 (MMP-2), growth/differentiation factor 15 (GDF-15), and interleukin 29 (IL-29). Screening for microvascular complications, such as autonomic and peripheral neuropathy, diabetic kidney disease, and retinopathy, was conducted. The group was divided according to the occurrence of microvascular complications. At least one complication was required for the patient to be included in the microangiopathy group. The median EGF concentration in the microangiopathy group was higher than in the group without microangiopathy (*p* = 0.03). Increasing EGF concentration was a statistically significant predictor of the presence of microangiopathy in multivariate logistic regression analysis (*p* < 0.0001). Additionally, a higher GDF-15 level was associated with diabetic kidney disease, peripheral neuropathy, and proliferative retinopathy vs. nonproliferative retinopathy. GDF-15 concentration correlated negatively with estimated glomerular filtration rate (eGFR) (r = −0.28; *p* = 0.02). To conclude, higher EGF concentration is an independent predictor of the presence of microvascular complications in T1DM patients. Besides the relation between GDF-15 and diabetic kidney disease, it may be also associated with peripheral neuropathy and retinopathy.

## 1. Introduction

Type 1 diabetes is an autoimmune disease frequently associated with microangiopathic complications [1]. Diabetic kidney disease, retinopathy, and peripheral and autonomic neuropathy are among the microangiopathic complications. To maintain a high quality of life and similar life expectancy to the normal population, early detection of complications is crucial in type 1 diabetes mellitus (T1DM) patients. Despite very important progress in the control of the disease and novel therapeutic options, the occurrence of neurovascular complications still cannot be fully prevented. Therefore, new markers of microangiopathy are being searched for intensely.

In the pathogenesis of diabetic complications development, processes affect actually existing vessels as well as the proliferation of new vessels. This is regulated via growth factors such as vascular endothelial growth factor (VEGF). However, there are publications questioning its main role [2]. The formation of new vessels, as well as a dysfunctional endothelium by itself, results in the secretion of inflammatory mediators such as tumor necrosis factor (TNF-α) and interleukin-6 (IL-6) [3]. There has been progress in knowledge thanks to studies focusing on glucotoxicity not only in endothelial cells but also in neurons (similar mechanisms related to oxidative stress were induced by intracellular high glucose concentration in the response of hyperglycemia). It has been proved that the neurodegenerative process is involved not only in the development of diabetic neuropathy but also in retinopathy. Altogether, microvascular complications have recently been called neurovascular complications [4]. There is a need to discover new factors promoting proper angiogenesis and inhibiting destructive processes, which may lead to the invention of new medications protecting against the development of chronic neurovascular complications.

Some proteins are interesting in their biological functions and can be engaged in the development of microangiopathy. What is more, no research has yet been conducted to explore them. One of them is epidermal growth factor (EGF), which is secreted by many types of cells such as platelets, macrophages, and fibroblasts. It activates its own receptors to promote cell proliferation and wound healing [5]. Preliminary studies have shown that EGF can be associated with the pathogenesis of microvascular complications. However, the majority of studies were conducted on mice and cell cultures, and it has been demonstrated that T1DM patients have higher blood concentrations of EGF as compared with healthy controls [6]. It has already been found that activated EGF receptors promote retinal dysfunction and vascular abnormalities in mouse models of diabetic retinopathy [7]. Studies on T1DM patients have not been carried out to assess the connection between EGF and microangiopathy.

Growth/differentiation factor 15 (GDF-15) is a member of the transforming growth factor β (TGF-β) superfamily. It is secreted in the course of cardiovascular disorders [8]. Besides its role in heart diseases, there are preliminary studies concerning the role of GDF-15 in diabetic kidney disease development. Higher GDF-15 concentrations were associated with a faster deterioration of kidney function but not with the development of end-stage renal disease [9,10]. Moreover, genetic deletion of the GDF-15 gene resulted in renal damage in mice models of type 1 and type 2 diabetes [11]. This suggests its probable nephroprotective role. Based on the above-mentioned publications, we have a strong premise to expect an association between GDF-15 and microvascular changes, but no study has yet assessed its concentration with regard to all complications of the disease.

Matrix metalloproteinases are a group of enzymes whose main task is the degradation of extracellular matrix components (e.g., collagen, elastin, gelatin, laminin) [12]. Matrix metalloproteinase 2 (MMP-2) is a gelatinase, also known as gelatinase A. This enzyme was an object of research in T1DM patients, and in comparison with healthy controls, its concentration was higher in T1DM subjects [2]. In microvascular disease, MMP-2 concentration has also been assessed. Patients with microalbuminuria vs. patients with normal urine test results, and proliferative vs. nonproliferative retinopathy, had higher MMP-2 concentrations [13]. However, the results of another study are contradictory [2]. Thus, there is still no clear evidence that MMP-2 might be a marker of neurovascular disease in T1DM patients.

Interleukin 29 (IL-29, also known as interferon lambda 1, IFN-λ1) is a new member of the recently discovered interferon lambda (IFN-λ) family. Dendritic cells and macrophages produce it as a response to autoimmune processes or viral infections [14]. Elevated Il-29 levels were detected in the following autoimmune disorders: Sjögren syndrome, rheumatoid arthritis, systemic sclerosis, and psoriasis [15,16,17,18]. Increased concentrations of this interleukin were also found in atopic dermatitis and asthma [19,20]. The literature data about this newly discovered protein are very limited. To the best of the authors’ knowledge, this is the first research work aiming to assess the concentration of this protein in T1DM patients.

Neurovascular complications still cannot be easily detected at an early stage. Therefore, this study has attempted to broaden our knowledge about their determinants by assessment of serum concentrations of EGF, MMP-2, GDF-15, and Il-29 and their relationships with the prevalence of microangiopathy in adult T1DM patients.

## 2. Materials and Methods

### 2.1. Study Population

This study was designed as a cross-sectional study. All experimental protocols conformed to the ethical guidelines of the 1975 Declaration of Helsinki concerning research involving human subjects and were approved by the Bioethical Committee of Poznan University of Medical Sciences (reference numbers: 173/17, 174/17, and 87/19). Every participant was involved voluntarily and provided written informed consent. The data that support the findings of this study are available from the corresponding author upon reasonable request.

A cohort of T1DM adults with at least 5 years duration of the disease was recruited in the Department of Internal Medicine and Diabetology in Poznan. Detailed inclusion and exclusion criteria are presented in Table 1.

The final study sample included 100 consecutive patients, of which 53 (53%) were males. Every patient was treated with intensive functional insulin therapy before admission to the hospital. Full and detailed baseline characteristics are presented in Table 2 for EGF, in Supplementary Table S1 for GDF-15 and MMP-2, and in Supplementary Table S2 for IL-29. Patients were selected randomly from a group of 100 consecutively enrolled patients to measure GDF-15, MMP-2, and IL-29. The whole group of 100 patients had EGF assessed.

The number of 100 participants was calculated based on assumption that each patient with microangiopathy will have elevated markers. We assumed based on our experience that 30% of participants will have at least one neurovascular complication (and simultaneously elevated markers) and 5% of patients without microangiopathy will have elevated markers. Enrollment ratio was 2:1 in calculation (about 30% with complications, 70% without), alpha 0.05, and power 0.9 [21,22].

Diagnosis of diabetes was made at least 5 years before study enrolment, and then American Diabetes Association criteria must have been met: classic symptoms of hyperglycemia or hyperglycemic crisis, and random plasma glucose concentration of ≥11.1 mmol/L [23]. Autoimmunology was confirmed by detecting at least one out of three T1DM-specific autoantibodies against islet cells (ICA), glutamic acid decarboxylase (GAD), and tyrosine phosphatase (IA-2).

### 2.2. Laboratory Assessment

Blood samples were obtained using a standard venipuncture between 06:00 and 10:00 after at least 8 h of overnight fasting. The concentrations in serum of the following proteins were measured: EGF, GDF-15, MMP-2, and IL-29. Moreover, every patient had the following laboratory tests performed on serum: alanine transaminase (ALT), aspartate transaminase (AST), estimated glomerular filtration rate (eGFR), glycated hemoglobin (in whole blood, HbA_1c_), high-density lipoprotein (HDL), high-sensitivity C-reactive protein (hsCRP), low-density lipoprotein (LDL), total cholesterol (TC), and triglycerides (TG).

HbA_1c_ was determined by turbidimetric inhibition immunoassay (TINIA) on a Cobas 6000 analyzer (Roche Diagnostics, Basel, Switzerland), and other measurements were performed using enzymatic assays on a Cobas 6000 analyzer. Estimated glomerular filtration rate (eGFR) was calculated afterwards using The Chronic Kidney Disease Epidemiology Collaboration (CKD-EPI) equation, which takes into account creatinine concentration in serum, age, and sex [24].

EGF, GDF-15, MMP-2, and IL-29 were measured by the EGF ELISA Kit (an intra-assay coefficient of variation (CV) < 10% and inter-assay CV < 10%), GDF15 ELISA Kit (intra-assay CV < 5.3%, inter-assay CV < 8.2%), MMP-2 ELISA Kit (intra-assay CV < 5.7%, inter-assay CV < 6.6%), and IL-29 ELISA Kit (intra-assay CV < 5.5%, inter-assay CV < 6.5%), respectively (Aviva Systems Biology, San Diego, CA, USA), which are enzyme immunoassays for quantitative in vitro laboratory measurements.

Laboratory analyses were performed on the same day, except concentrations of EGF, GDF-15, MMP-2, and IL-29, which were measured from the serum, previously stored frozen at −80 °C, after all samples were collected.

### 2.3. Evaluation of Neurovascular Complications

Patients were divided into two groups according to the presence or absence of microangiopathy. At least one of the following complications qualified a patient to the group with microangiopathy: retinopathy, diabetic kidney disease, peripheral and autonomic neuropathy [25].

Diabetic kidney disease was diagnosed in the case of reduced eGFR in the absence of signs or symptoms of other primary causes of kidney damage, or after performing a 24 h urinary albumin excretion measurement (expressed in mg) or random albumin/creatinine ratio measurement (expressed in milligrams of albumin per gram of creatinine (mg/g)). Excessive physical activity, infections, heart failure, and hematuria constituted exclusion criteria. Normoalbuminuria was defined as 24 h urinary albumin excretion lower than 30 mg/24 h or albumin/creatinine ratio lower than 30 mg/g [26]. A higher value of albumin/creatinine ratio was considered as abnormal. To make a diagnosis of diabetic kidney disease, two subsequent abnormal urine test results were required over 10 years of T1DM duration or confirmed diabetic retinopathy. In some cases, diagnosis was made before the study enrolment and information was based on medical history.

Ophthalmological examination was performed using direct ophthalmoscopy through dilated pupils according to the American Academy of Ophthalmology guidelines. The following categories of result were applied in the current study: no retinopathy, nonproliferative retinopathy, and proliferative retinopathy [27].

The diagnosis of diabetic peripheral neuropathy was based on American Diabetes Association diagnostic criteria [28]. Two or more out of the following criteria were required to diagnose diabetic peripheral neuropathy: symptoms reported by the patient, abnormal touch sensation (evaluated with the Semmes–Weinstein 10 g monofilament at many sites on each foot), abnormal feeling of vibration (128 Hz tuning fork), abnormal temperature sensation (TipTherm), abnormal Achilles tendon reflexes.

Cardiac autonomic neuropathy was assessed clinically with the validated ProSciCard III program (CPS GmbH, Wetzlar, Germany, 2010) [29,30].

### 2.4. Assessment of Other Variables

Every participant underwent a basic physical examination that included blood pressure assessment and anthropometric measurements. Hypertension was assessed as present if the patient reported so or used antihypertensive medications or was newly diagnosed (by aneroid sphygmomanometer after at least 10 min rest in a sitting position). Arterial hypertension was defined as a mean value of systolic blood pressure (SBP) ≥ 140 mmHg and/or diastolic blood pressure (DBP) ≥ 90 mmHg. Body mass index (BMI) was calculated using the following formula: BMI = weight/height^2^ [kg/m^2^].

Data on sociodemographic characteristics and medical history were collected via questionnaire.

### 2.5. Statistical Analysis

The acquired data were analyzed using Statistica V13 software (StatSoft, Tulsa, OK, USA, RRID:SCR_014213, http://www.statsoft.com). First, a Shapiro–Wilk test was applied to assess whether qualitative variables were normally distributed. The distributions of all the qualitative variables were not normal, thus nonparametric statistical tests were applied. Qualitative variables are expressed as median and interquartile range (IQR), and categorical variables as number (%) of subjects. EGF concentration is also presented as Log10EGF (decadic logarithm) because of the normality required in multivariate linear regression analysis. In descriptive characteristics, comparisons of parameters on dichotomous scales were prepared using a chi-square test, and for the remaining variables, which were continuous, a Mann–Whitney U test was applied. The Spearman’s rank correlation coefficients were calculated to estimate the correlations between selected parameters. A multivariate logistic regression model was built to examine the relationships between EGF and the prevalence of neurovascular complications. Additionally, EGF was also evaluated in terms of receiver operating characteristic (ROC) curve with the Youden index to determine the cut-off. The *p*-value threshold was set at <0.05 for statistical significance.

## 3. Results

### 3.1. General Analysis

The median age of participants was 29 (IQR: 25–34.5) years, and median T1DM duration was 12.5 (IQR: 9–16) years. Full baseline characteristics are presented in Table 2 for EGF, in Supplementary Table S1 for GDF-15 and MMP-2, and in Supplementary Table S2 for IL-29. In the EGF study group, 32 (32%) participants were diagnosed with at least one neurovascular complication. The prevalence of complications was as follows: the most frequent was retinopathy at 30% (19% proliferative, 11% nonproliferative), while others, such as diabetic kidney disease at 8%, autonomic neuropathy at 9%, and peripheral neuropathy at 5%, occurred less frequently.

Participants in the subgroup with microangiopathy as compared with the subgroup free of these complications had longer disease duration (16 (14–18.5) vs. 11 (7–13.5) years; *p* < 0.001) and higher abnormal creatinine/albumin ratio prevalence (28% vs. 1%; *p* < 0.001). We found no differences in Il-29, GDF-15, and MMP-2 concentrations between these two subgroups.

### 3.2. EGF and Neurovascular Complications

Participants in the subgroup with microangiopathy as compared with subgroup free of these complications had higher EGF concentration (57.5 (IQR: 28.5–100.5) vs. 28.5 (IQR: 15–76) pg/mL; *p* = 0.032).

Correlations between selected proteins and other parameters are presented in Supplementary Table S3. Statistically significant positive correlations between EGF concentration and SBP (r = 0.21; *p* = 0.035) and GDF-15 concentration (r = 0.33; *p* = 0.007) were found.

In multivariate logistic regression analysis (Table 3), increasing EGF turned out to be statistically significantly associated with the presence of neurovascular complications (odds ratio (OR): 3.84; 95% confidence interval (CI): 1.04–14.11; *p* = 0.04) after an adjustment for T1DM duration, HbA1c, BMI, LDL, and SBP (*p* < 0.0001).

The ROC analysis for EGF at microangiopathy diagnosis revealed an EGF cut-off of 40 pg/mL (Youden method, Youden index 0.26; sensitivity 0.66 and specificity 0.60; positive likelihood ratio (LR+) 0.78 and negative likelihood ratio (LR−) 0.27) as the best value that significantly indicated the presence of neurovascular complications (area under the ROC curve (AUC): 0.636; 95% confidence interval (CI): 0.522–0.749; *p* = 0.019). A similar value for the crossing proportion point (cut-off 42 pg/mL; sensitivity 0.60 and specificity 0.61) was estimated.

### 3.3. GDF-15 and Neurovascular Complications

Patients with abnormal creatinine/albumin ratios had higher GDF-15 concentrations vs. patients with normal ratios (57 (43.5–64) vs. 23 (15.6–41) pg/mL; *p* = 0.002). As expected, the concentration of GDF-15 was also higher within the group of patients with diabetic kidney disease vs. patients free from this complication (59 (42–66) vs. 25.5 (15.6–41.5) pg/mL; *p* = 0.004). Similar to the above, patients with peripheral neuropathy had higher concentrations of GDF-15 vs. patients without it (62 (42–73) vs. 28.5 (15.6–43) pg/mL; *p* = 0.045). Moreover, patients with proliferative retinopathy had higher GDF-15 concentrations vs. patients with nonproliferative retinopathy (55 (30–66) vs. 15.6 (15.6–23) pg/mL; *p* = 0.023). Unfortunately, we found no statistically significant difference in GDF-15 concentration between patients with and without retinopathy, as well as with and without microangiopathy altogether.

GDF-15 concentration correlated positively with concentrations of hsCRP (r = 0.27; *p* = 0.027) and MMP-2 (r = 0.27; *p* = 0.029), and negatively with eGFR (r = −0.28; *p* = 0.02).

In multivariate logistic regression analysis (Supplementary Table S4), increasing GDF-15 turned out to be a statistically significant independent predictor of the prevalence of diabetic kidney disease (OR: 1.07; 95% CI: 1.01–1.12; *p* = 0.015) after an adjustment for T1DM duration, HbA1c, BMI, and smoking (*p* = 0.0002).

## 4. Discussion

The present study was conducted with the aim of searching for new determinants of neurovascular complications among selected novel inflammatory markers: EGF, MMP-2, GDF-15, and IL-29. The most valuable result of the current study is the revealed relationship between EGF and microangiopathy. In multivariate logistic regression analysis, this factor turned out to be a significant and independent predictor of neurovascular complications in T1DM patients. ROC analysis showed that EGF measurement can be especially used as a tool to exclude microangiopathic complications (LR− = 0.27). The value of EGF to affirm diagnosis of microangiopathy is low (LR+ = 0.78). Hence, it probably cannot be used to detect affected individuals, but it can be useful do detect individuals without microangiopathy.

Although it was previously revealed that blood concentration of EGF is higher among T1DM patients vs. healthy controls, further analyses were not conducted [6]. Urinary EGF was examined in a large cohort of 642 type 2 diabetic people to investigate its relationship with diabetic kidney disease [31]. Lower urinary EGF-to-creatinine ratio was directly related to deterioration of renal function in normoalbuminuric patients. Unfortunately, patients with diabetic kidney disease were not enrolled in the above-mentioned study. Hence, it can be only a clue indicating the relationship between EGF and renal function. Furthermore, the results of another study, where urinary EGF was measured in a cohort of individuals with eGFR < 60 mL/min/1.73 m^2^ and urinary albuminuria > 300 mg/24 h, revealed a lower concentration of urine EGF per gram of urine creatinine [32]. A study on mice demonstrated that EGF might be involved in diabetic kidney disease development as it is a growth factor also produced locally in distal tubular cells and it plays a significant role in the repair and regeneration of renal tubules [33]. Lower urine EGF may provide evidence for the depletion of repair mechanisms. There is no study evaluating an association between serum EGF and diabetic kidney disease. Only one study aimed to assess EGF in serum and revealed higher EGF levels in patients with diabetic kidney disease as compared with patients free of this complication [34]. Only one study revealed higher EGF serum concentration in a cohort of people with diabetic retinopathy as compared with controls without retinopathy [35]. However, T1DM patients were excluded from this study. On the other hand, another study did not confirm that conclusion [36]. It is postulated that, similarly to renal tubules, the retina also produces its own EGF [37]. Although EGF levels were also measured in the vitreous fluid of patients with proliferative diabetic retinopathy, the levels were very low or below the detection limit of the assay [38]. It is very probable that EGF takes part in local repair mechanisms that are depleted at some stage of the microvascular disease. Therefore, a higher EGF concentration can provide information about an intensive vascular repair process, but local production is too low to be detected. It is important to emphasize that EGF is produced in every endothelium and can be a marker of a damaged vessel. Also, atherosclerosis leads to an increase in EGF receptor expression [39]. Summarizing the above, EGF is probably connected with diabetic kidney disease. Other neurovascular complications were not assessed. The novelty of the current research is its conclusion on the potential role of EGF measured in serum as a universal marker of microangiopathy in T1DM patients.

In our study, GDF-15 turned out to be a statistically significant marker of diabetic kidney disease occurrence. It also correlated with eGFR. The most important study to compare with this result is a large and meticulously prepared prospective project named EURAGEDIC, conducted on T1DM patients [9]. In this study, patients with diabetic kidney disease had higher GDF-15 concentrations, similarly to our patients. Moreover, higher GDF-15 concentration was an independent predictor of kidney function deterioration and all-cause mortality in patients with diabetic kidney disease. In this study, eGFR also correlated negatively with GDF-15 level. The novel observation derived from the current research is that a higher concentration of GDF-15 was observed within the group with peripheral neuropathy vs. patients free from peripheral neuropathy. This is the first study reporting such a relation. Moreover, patients with proliferative retinopathy had higher GDF-15 concentrations as compared with patients with nonproliferative retinopathy. There is only one previous study aiming to assess GDF-15 concentration in patients with retinal disease with an inflammatory component. GDF-15 was measured in the retina, and an elevated concentration was found. Some patients were diabetic, but proliferative diabetic retinopathy was compared with rhegmatogenous retinal detachment, idiopathic epiretinal membranes, and macular holes, hence the study lacks a true control group. The biological targets of GDF-15 are tissues where EGF is expressed: cardiomyocytes, endothelial cells, and vascular smooth muscle cells. Its concentration is elevated during cell damage and inflammation [40]. There are no more studies concerning GDF-15 and microvascular disease in diabetes mellitus, but it is very possible that vascular inflammation results in increased GDF-15 production, which is revealed in the current study. To conclude, our results suggest that not only diabetic kidney disease but also other neurovascular complications are associated with the concentration of GDF-15. This very novel observation requires further and profound research.

In our study, we did not obtain any relation between MMP-2 and diabetic microangiopathy. This lack of relation between MMP-2 and microvascular changes is consistent with the available literature. MMP-2 was previously an object of several studies in T1DM patients. The most important study in this matter was conducted by Peeters et al. in a cohort of 493 T1DM subjects [13]. In this study, where there were higher plasma levels of MMP-2, higher levels of albuminuria were detected. Additionally, more severe retinopathy was detected in patients with higher MMP-2 concentrations. Unfortunately, no relation at all between MMP-2 and microangiopathy was found in the above-mentioned study. Higher concentrations of MMP-2 in diabetic vs. healthy subjects were described in several studies [2,41]. However, these studies were performed on small cohorts and did not present results consistent to Peeters et al. [13]. In one study, an even lower level of MMP-2 in diabetic nephropathy vs. diabetes without nephropathy was described [42]. Thrailkill et al. also did not perform controlled examinations of retinopathy and neuropathy, which were only assessed via questionnaire. MMP-2 was also previously investigated in the aqueous humor of the eyes of patients with diabetic retinopathy, and increased activity was detected in patients with diabetic retinopathy vs. patients free from this complication [43]. There are also studies indicating that increased expression of MMP-2 exists in experimental models of renal injury during ischemia [44]. However, the exact mechanism is not a result of small vessel disease, so based on the results of our study and previous research, it seems that MMP-2 is not an important factor influencing microvascular changes in T1DM patients.

Another protein investigated in the current study was Il-29. Unfortunately, it turned out not to be associated with the clinical parameters and prevalence of neurovascular complications. Probably, it is associated with an acute autoimmune process that is not present in patients with a disease duration of longer than 5 years, as was the case in our cohort. Moreover, inflammation within the small vessels in the course of diabetes is very subtle and does not affect the whole organism as it does during acute viral infections. Thus, although other interleukins such as IL-6 were found to be related to diabetes, Il-29 seems not to take part in microvessel damage in the course of diabetes.

We must also address the mutual correlations between the pairs EGF/GDF-15 and GDF-15/MMP-2. As they are inflammation-related proteins, even local and chronic inflammation can result in low correlation. GDF-15 and EGF are probably associated with microangiopathy. MMP-2 is also described as related to diabetic kidney disease, which was not reflected in the current study. However, it was correlated with GDF-15 concentrations, which are related to diabetic kidney disease, so it can be a reflection of such evidence.

Finally, several limitations of our study must be emphasized, including the fact that it was a single-center trial and the limited size of the studied group. Another limitation is measurement of GDF-15, MMP-2, and IL-29 not on all subjects. The whole group of 100 patients had EGF assessed. Moreover, this study does not establish any causal link between the dysregulation of the investigated proteins and the development of diabetic complications, as the conclusions on the relation were made only on the basis of single-point measurements. We also cannot indicate tissue sources of examined proteins. Our observation may become a useful background to plan a prospective study evaluating the relationship between proteins such as EGF and GDF-15 and the prevalence of microvascular disease within a population of patients with T1DM.

## 5. Conclusions

To conclude, impacts of EGF and GDF-15 on the prevalence of neurovascular complications in the course of T1DM are very possible. The results strongly indicate that in T1DM, serum EGF concentration might serve as a useful marker to detect microangiopathy, while GDF-15 may probably be engaged to some extent in microvascular damage as a marker of diabetic kidney disease, peripheral neuropathy, and retinopathy. However, further and prospective studies on larger groups of patients should be conducted in the future to assess the full clinical utility of these markers.

## Figures and Tables

**Table 1:** Inclusion and exclusion criteria.

Inclusion criteria	European Caucasian origin Type 1 diabetes Age between 18 and 50 years Duration of the disease ≥ 5 years
Exclusion criteria	eGFR below 30 mL·min^−1^·1.73 m^−2^ ALT or AST 1.5 times above upper limit of normal Acute inflammation (hsCRP > 10 mg/L or symptoms) Diabetic ketoacidosis or ketonuria Neoplasm

Abbreviations: ALT, alanine transaminase; AST, aspartate transaminase; eGFR, estimated glomerular filtration rate; hsCRP, high-sensitivity C-reactive protein.

**Table 2:** Characteristics of the study participants according to the presence of microangiopathy in the EGF cohort (number or median (IQR)).

Parameter	Value			
	All Participants (*n* = 100)	With Microangiopathy (*n* = 32)	Without Microangiopathy (*n* = 68)	*p* *
Age, years	29 (25–34.5)	29 (25.5–34.5)	29.5 (25–34.5)	0.695
Men, n (%)	53 (53.0)	19 (59.4)	34 (50.0)	0.381 **
Smokers, n (%)	18 (18.0)	9 (28.1)	9 (13.2)	0.071 **
T1DM duration, years	12.5 (9–16)	16 (14–18.5)	11 (7–13.5)	<0.001
Abnormal creatinine/albumin ratio, n (%)	10 (10)	9 (28)	1 (1)	<0.001 ***
SBP, mmHg	125 (120–130)	130 (120–130)	120 (120–130)	0.449
DBP, mmHg	80 (70–83)	80 (80–90)	80 (70–80)	0.073
BMI, kg/m^2^	24.1 (22.1–26.8)	24.6 (21.9–27.5)	24.1 (22.2–26.4)	0.851
HbA_1c_, %	7.4 (6.8–8.3)	7.55 (6.95–8.1)	7.35 (6.7–8.3)	0.900
hsCRP, mg/L	1.08 (0.35–2.51)	1.42 (0.66–2.34)	0.97 (0.34–2.76)	0.363
ALT, U/L	17 (13–21)	18.5 (13–26)	15.5 (13–21)	0.213
AST, U/L	17 (15–20)	18.5 (15–22)	17 (14–20)	0.273
TC, mmol/L	4.69 (4.09–5.37)	4.84 (4.38–5.26)	4.65 (4.00–5.54)	0.739
HDL, mmol/L	1.68 (1.40–1.92)	1.70 (1.37–1.98)	1.68 (1.45–1.92)	0.770
LDL, mmol/L	2.78 (2.23–3.38)	2.68 (2.34–3.17)	2.82 (2.15–3.52)	0.787
TG, mmol/L	0.97 (0.70–1.27)	1.07 (0.87–1.66)	0.91 (0.66–1.20)	0.058
eGFR, mL·min^−1^·1.73 m^−2^	109 (96–118)	102 (89–119)	109 (99–117)	0.233
EGF, pg/mL	36.5 (16–81.5)	57.5 (28.5–100.5)	28.5 (15–76)	0.032
Log_10_EGF	1.56 (1.20–1.91)	1.75 (1.45–2.00)	1.45 (1.18–1.88)	0.028

* *p*-value for comparison of groups in accordance with microangiopathy occurrence; ** *χ*^2^ test; *** Yates-corrected *χ*^2^ test; Mann–Whitney U test in every other case, where it is not marked with ** or ***. Abbreviations: ALT, alanine transaminase; AST, aspartate transaminase; BMI, body mass index; DBP, diastolic blood pressure; EGF, epidermal growth factor concentration; eGFR, estimated glomerular filtration rate; HbA_1c_, glycated hemoglobin; HDL, high-density lipoprotein; hsCRP, high-sensitivity C-reactive protein; IQR, interquartile range; LDL, low-density lipoprotein; Log_10_EGF, decadic logarithm of epidermal growth factor concentration; SBP, systolic blood pressure; T1DM, type 1 diabetes; TC, total cholesterol; TG, triglycerides; WHR, waist-to-hip ratio. A *p*-value less than 0.05 was considered statistically significant. The bolded *p*-values are those which are statistically significant.

**Table 3:** Markers of the presence of neurovascular complications occurrence in multivariate logistic regression analysis, with microangiopathy as the dependent variable and Log_10_EGF, T1DM duration, HbA_1c_, BMI, LDL, and SBP as independent variables. For the entire model, *p* < 0.0001.

Predictors	Odds Ratio (95% Confidence Interwal)	*p*-Value
Log_10_EGF	3.84 (1.04–14.11)	**0.040**
T1DM duration	1.27 (1.13–1.44)	**<0.0001**
HbA_1c_	1.05 (0.66–1.66)	0.829
BMI	0.98 (0.85–1.13)	0.749
LDL	1.06 (0.57–1.96)	0.859
SBP	0.99 (0.95–1.04)	0.828

Abbreviations: see Table 2. The bolded *p*-values are those which are statistically significant.

## Supplementary Materials

The following are available online at https://www.mdpi.com/2077-0383/9/1/198/s1, Supplementary Table S1. Characteristics of the study participants according to the presence of microangiopathy in the GDF-15 and MMP-2 cohort (number or median (IQR)), Supplementary Table S2. Characteristics of the study participants according to the presence of microangiopathy in the IL-29 cohort (number or median (IQR)), Supplementary Table S3. Correlations between various parameters and EGF concentration (Spearman’s rank correlation analysis), Supplementary Table S4. Markers of diabetic kidney disease occurrence in multivariate logistic regression analysis, with diabetic kidney disease as the dependent variable and GDF-15, T1DM duration, HbA_1c_, smoking, and BMI as independent variables. For the entire model, *p* = 0.0002.

## References

[1] Wang G., Yan Y., Xu N., Yin D., Hui Y. (2019). Treatment of type 1 diabetes by regulatory T-cell infusion via regulating the expression of inflammatory cytokines. J. Cell Biochem..

[2] Thrailkill K.M., Bunn R.C., Moreau C.S., Cockrell G.E., Simpson P.M., Coleman H.N., Frindik J.P., Kemp S.F., Fowlkes J.L. (2007). Matrix metalloproteinase-2 dysregulation in type 1 diabetes. Diabetes Care.

[3] Wegner M., Araszkiewicz A., Piorunska-Stolzmann M., Wierusz-Wysocka B., Zozulinska-Ziolkiewicz D. (2013). Association between IL-6 concentration and diabetes-related variables in DM1 patients with and without microvascular complications. Inflammation.

[4] Araszkiewicz A., Zozulinska-Ziolkiewicz D. (2016). Retinal Neurodegeneration in the Course of Diabetes-Pathogenesis and Clinical Perspective. Curr. Neuropharmacol..

[5] Yang Q., Zhang Y., Yin H., Lu Y. (2019). Topical recombinant human epidermal growth factor for diabetic foot ulcers: A meta-analysis of randomized controlled clinical trials. Ann. Vasc. Surg..

[6] Sochett E., Noone D., Grattan M., Slorach C., Moineddin R., Elia Y., Mahmud F.H., Dunger D.B., Dalton N., Cherney D. (2017). Relationship between serum inflammatory markers and vascular function in a cohort of adolescents with type 1 diabetes. Cytokine.

[7] Ju X., Yang X., Yan T., Chen H., Song Z., Zhang Z., Wu W., Wang Y. (2019). EGFR inhibitor, AG1478, inhibits inflammatory infiltration and angiogenesis in mice with diabetic retinopathy. Clin. Exp. Pharmacol. Physiol.

[8] Kempf T., Kempf T., Eden M., Strelau J., Naguib M., Willenbockel C., Tongers J., Heineke J., Kotlarz D., Xu J. (2006). The transforming growth factor-beta superfamily member growth-differentiation factor-15 protects the heart from ischemia/reperfusion injury. Circ. Res..

[9] Lajer M., Jorsal A., Tarnow L., Parving H.H., Rossing P. (2010). Plasma growth differentiation factor-15 independently predicts all-cause and cardiovascular mortality as well as deterioration of kidney function in type 1 diabetic patients with nephropathy. Diabetes Care.

[10] Hellemons M.E., Mazagova M., Gansevoort R.T., Henning R.H., de Zeeuw D., Bakker S.J.L., Lambers-Heerspink H.J., Deelman L.E. (2012). Growth-differentiation factor 15 predicts worsening of albuminuria in patients with type 2 diabetes. Diabetes Care.

[11] Mazagova M., Buikema H., van Buiten A., Duin M., Goris M., Sandovici M., Henning R.H., Deelman L.E. (2013). Genetic deletion of growth differentiation factor 15 augments renal damage in both type 1 and type 2 models of diabetes. Am. J. Physiol. Renal. Physiol..

[12] Visse R., Nagase H. (2003). Matrix metalloproteinases and tissue inhibitors of metalloproteinases: Structure, function, and biochemistry. Circ. Res..

[13] Peeters S.A., Hanada H., Ishizaka H., Fukushi T., Kamada T., Okumura K. (2015). Plasma levels of matrix metalloproteinase-2, -3, -10, and tissue inhibitor of metalloproteinase-1 are associated with vascular complications in patients with type 1 diabetes: The EURODIAB Prospective Complications Study. Cardiovasc. Diabetol..

[14] Lazear H.M., Nice T.J., Diamond M.S. (2015). Interferon-lambda: Immune Functions at Barrier Surfaces and Beyond. Immunity.

[15] Ha Y.J., Choi Y.S., Kang E.H., Chung J.H., Cha S., Song Y.W., Lee Y.J. (2018). Increased expression of interferon-lambda in minor salivary glands of patients with primary Sjogren’s syndrome and its synergic effect with interferon-alpha on salivary gland epithelial cells. Clin. Exp. Rheumatol..

[16] Chang Q.J., Lv C., Zhao F., Xu T.S., Li P. (2017). Elevated Serum Levels of Interleukin-29 Are Associated with Disease Activity in Rheumatoid Arthritis Patients with Anti-Cyclic Citrullinated Peptide Antibodies. Tohoku J. Exp. Med..

[17] Dantas A.T., Gonçalves S.M.C., Pereira M.C., de Almeida A.R., Marques C.D.L., Rego M.J.B.D., Pitta I.D., Duarte A.L.B.P., Pitta M.G.D. (2015). Interferons and systemic sclerosis: Correlation between interferon gamma and interferon-lambda 1 (IL-29). Autoimmunity.

[18] Wolk K., Witte K., Witte E., Raftery M., Kokolakis G., Philipp S., Schönrich G., Warszawska K., Kirsch S., Prösch S. (2013). IL-29 is produced by T(H)17 cells and mediates the cutaneous antiviral competence in psoriasis. Sci. Trans. Med..

[19] Da Silva J., Hilzendeger C., Moermans C., Schleich F., Henket M., Kebadze T., Mallia P., Edwards M.R., Johnston S.L., Louis R. (2017). Raised interferon-beta, type 3 interferon and interferon-stimulated genes—Evidence of innate immune activation in neutrophilic asthma. Clin. Exp. Allergy.

[20] Fedenko E.S., Elisyutina O.G., Filimonova T.M., Boldyreva M.N., Burmenskaya O.V., Rebrova O.Y., Yarilin A.A., Khaito R.M. (2011). Cytokine gene expression in the skin and peripheral blood of atopic dermatitis patients and healthy individuals. Self.

[21] Association A.D. (2018). 2. Classification and Diagnosis of Diabetes: Standards of Medical Care in Diabetes-2018. Diabetes Care.

[22] Rosner B. (2011). Fundamentals of Biostatistics.

[23] ClinCalc.com https://clincalc.com/stats/samplesize.aspx.

[24] Levey A.S., Stevens L.A., Schmid C.H., Zhang Y., Castro A.F., Feldman H.I., Kusek J.W., Eggers P., van Lente F., Greene T. (2009). A new equation to estimate glomerular filtration rate. Ann. Intern. Med..

[25] Falkowski B., Rogowicz-Frontczak A., Grzelka A., Uruska A., Schlaffke J., Araszkiewicz A., Zozulinska-Ziolkiewicz D. (2018). Higher free triiodothyronine concentration is associated with lower prevalence of microangiopathic complications and better metabolic control in adult euthyroid people with type 1 diabetes. Endocrine.

[26] Yang X.H., Feng S.Y., Yu Y., Liang Z. (2018). Study on the relationship between the methylation of the MMP-9 gene promoter region and diabetic nephropathy. Endokrynol. Pol..

[27] Mrozikiewicz-Rakowska B., Łukawska M., Nehring P., Szymański K., Sobczyk-Kopcioł A., Krzyżewska M., Maroszek P., Płoski R., Czupryniak L. (2018). Genetic predictors associated with diabetic retinopathy in patients with diabetic foot. Pol. Arch. Intern. Med..

[28] Association A.D. (2020). 11. Microvascular Complications and Foot Care: Standards of Medical Care in Diabetes-2020. Diabetes Care.

[29] Ziegler D., Laux G., Dannehl K., Spüler M., Mühlen H., Mayer P., Gries F.A. (1992). Assessment of cardiovascular autonomic function: Age-related normal ranges and reproducibility of spectral analysis, vector analysis, and standard tests of heart rate variation and blood pressure responses. Diabetes Med..

[30] Pawlinski L., Gastol J., Fiema M., Matejko B., Kiec-Wilk B. (2019). Is our treatment in type 1 diabetes mellitus (insulin therapy models, metabolic control) optimal for preventing cardiovascular autonomic neuropathy?. Endokrynol. Pol..

[31] Betz B.B., Jenks S.J., Cronshaw A.D., Lamont D.J., Cairns C., Manning J.R., Goddard J., Webb D.J., Mullins J.J., Hughes J. (2016). Urinary peptidomics in a rodent model of diabetic nephropathy highlights epidermal growth factor as a biomarker for renal deterioration in patients with type 2 diabetes. Kidney Int..

[32] De Boer I.H., Gao X., Bebu I., Hoofnagle A.N., Lachin J.M., Paterson A., Perkins B.A., Saenger A.K., Steffes M.W., Zinman B. (2017). Biomarkers of tubulointerstitial damage and function in type 1 diabetes. BMJ Open Diabetes Res. Care.

[33] Chen J., Harris R.C. (2016). Interaction of the EGF Receptor and the Hippo Pathway in the Diabetic Kidney. J. Am. Soc. Nephrol..

[34] Perlman A.S., Chevalier J.M., Wilkinson P., Liu H., Parker T., Levine D.M., Sloan B.J., Gong A., Sherman R., Farrell F.X. (2015). Serum Inflammatory and Immune Mediators Are Elevated in Early Stage Diabetic Nephropathy. Ann. Clin. Lab. Sci..

[35] Malik T.G., Ahmed S.S., Gul R., Ayesha E. (2018). Comparative Analysis of Serum Proangiogenic Biomarkers between those with and without Diabetic Retinopathy. J. Coll. Physicians Surg..

[36] Ozturk B.T., Bozkurt B., Kerimoglu H., Okka M., Kamis U., Gunduz K. (2009). Effect of serum cytokines and VEGF levels on diabetic retinopathy and macular thickness. Mol. Vis..

[37] Patel B., Hiscott P., Charteris D., Mather J., McLeod D., Boulton M. (1994). Retinal and preretinal localisation of epidermal growth factor, transforming growth factor alpha, and their receptor in proliferative diabetic retinopathy. Br. J. Ophthalmol..

[38] Boulton M., Gregor Z., McLeod D., Charteris D., Jarvis-Evans J., Moriarty P., Khaliq A., Foreman D., Allamby D., Bardsley B. (1997). Intravitreal growth factors in proliferative diabetic retinopathy: Correlation with neovascular activity and glycaemic management. Br. J. Ophthalmol..

[39] Lamb D.J., Modjtahedi H., Plant N.J., Ferns G.A. (2004). EGF mediates monocyte chemotaxis and macrophage proliferation and EGF receptor is expressed in atherosclerotic plaques. Atherosclerosis.

[40] Adela R., Banerjee S.K. (2015). GDF-15 as a Target and Biomarker for Diabetes and Cardiovascular Diseases: A Translational Prospective. J. Diabetes Res..

[41] Shiau M.Y., Tsai S.T., Tsai K.J., Haung M.L., Hsu Y.T., Chang Y.H. (2006). Increased circulatory MMP-2 and MMP-9 levels and activities in patients with type 1 diabetes mellitus. Mt. Sinai J. Med..

[42] Rysz J., Banach M., Stolarek R.A., Pasnik J., Cialkowska-Rysz A., Koktysz R., Piechota M., Baj Z. (2007). Serum matrix metalloproteinases MMP-2 and MMP-9 and metalloproteinase tissue inhibitors TIMP-1 and TIMP-2 in diabetic nephropathy. J. Nephrol..

[43] Klysik A.B., Naduk-Kik J., Hrabec Z., Gos R., Hrabec E. (2010). Intraocular matrix metalloproteinase 2 and 9 in patients with diabetes mellitus with and without diabetic retinopathy. Arch. Med. Sci..

[44] Dejonckheere E., Vandenbroucke R.E., Libert C. (2011). Matrix metalloproteinases as drug targets in ischemia/reperfusion injury. Drug Discov. Today.

