# Supplementary material for: Novel Biochemical Markers of Neurovascular Complications in Type 1 Diabetes Patients
Source: J Clin Med. 2020 Jan 10;9(1):198. doi: 10.3390/jcm9010198 (PMC7027005; doi:10.3390/jcm9010198)
Supplement: Supplementary file 1 [file jcm-09-00198-s001.pdf]

Supplementary Table 1. Characteristics of the study participants according to the presence of microangiopathy in the GDF-15 and MMP-2 cohort [number or median (IQR)].

**Parameter**	**Value**			
	**All participants (n=69)**	**With microangiopathy (n=19)**	**Without microangiopathy (n=50)**	**P***
Age, years	30 (26–34)	29 (25–33)	31 (27–35)	0.390
Men, n (%)	38 (55.0)	12 (63.1)	26 (52.0)	0.405**
Smoker, n (%)	11 (15.9)	5 (26.3)	6 (12.0)	0.147**
T1DM duration, years	13 (9–16)	17 (14–19)	11 (7–14)	**<0.001**
Abnormal creatinine/albumin ratio, n (%)	8 (11.6)	7 (36.8)	1 (2.0)	**<0.001*** **
SBP, mmHg	130 (120–130)	130 (120–130)	125 (120–130)	0.498
DBP, mmHg	80 (70–80)	80 (80–82)	80 (70-80)	0.214
BMI, kg/m^2	24.1 (22.2–26.4)	24.1 (21.5–27.5)	23.9 (22.2–26.1)	0.697
HbA_{1c}, %	7.3 (6.7–8.1)	7.2 (6.8–8.1)	7.3 (6.65–8.3)	0.762
hsCRP, mg/L	0.73 (0.30–1.60)	0.89 (0.32–1.76)	0.70 (0.29–1.47)	0.357
ALT, U/L	17 (12–21)	20 (16–23)	15 (12–20)	0.111
AST, U/L	17 (15–20)	19.5 (15–22)	17 (15–20)	0.159
TC, mmol/L	4.69 (4.09–5.31)	4.92 (4.4–5.23)	4.58 (4.04–5.36)	0.498
HDL, mmol/L	1.68 (1.4–1.92)	1.68 (1.37–2.02)	1.67 (1.40–1.92)	0.814
LDL, mmol/L	2.85 (2.28–3.39)	2.9 (2.43–3.24)	2.82 (2.18–3.55)	0.898
TG, mmol/L	0.90 (0.65–1.15)	1.00 (0.84–1.25)	0.84 (0.60–1.12)	0.037
eGFR, mL·min^{-1}·1.73m^{-2}	107 (96–115)	101 (89–117)	109 (98–115)	0.268
GDF-15, pg/mL	29 (15.6–45)	28 (15.6–55)	29.5 (15.6–43)	0.757
MMP-2, ng/mL	9.7 (8.4–11.4)	9.3 (8.4–11.7)	10.15 (7.8–11.4)	0.968

* P value for comparison groups in accordance to microangiopathy occurrence ** χ^2 test; *** Yates corrected χ^2 test; Mann–Whitney U test in every other case, where it is not marked with **. Abbreviations: GDF-15, growth/differentiation factor 15; MMP-2, metalloproteinase 2 concentration; other see Table 2. A P value less than 0.05 was considered statistically significant. The bolded p-values are those which are statistically significant.

Supplementary Table 2. Characteristics of the study participants according to the presence of microangiopathy in the IL-29 cohort [number or median (IQR)].

Parameter	Value			
	**All participants (n=80)**	**With microangiopathy (n=30)**	**Without microangiopathy (n=50)**	**P***
Age, years	29 (25.5–34)	29 (26–34)	29 (25–34)	0.785
Men, n (%)	44 (55.0)	18 (60.0)	26 (52.0)	0.486**
Smoker, n (%)	14 (17.5)	8 (26.7)	6 (12.0)	0.094**
T1DM duration, years	13 (9–16)	16 (14–19)	11 (7-13)	**<0.001**
Abnormal creatinine/albumin ratio, n (%)	9 (11.3)	8 (26.7)	1 (2.0)	**0.003*** **
SBP, mmHg	130 (120–130)	130 (120–130)	125 (120–130)	0.574
DBP, mmHg	80 (78.5–85)	80 (80–90)	80 (70–80)	0.214
BMI, kg/m^2	24.2 (21.7–26.6)	24.3 (21.5–27.4)	24.2 (21.7–26.4)	0.743
HbA_{1c}, %	7.49 (6.8–8.3)	7.75 (7–8.1)	7.43 (6.7–8.3)	0.691
hsCRP, mg/L	0.96 (0.36–2.15)	1.29 (0.65–2.11)	0.76 (0.36–2.19)	0.531
ALT, U/L	17 (13–21.5)	18.5 (12.5–26.5)	17 (13–20.5)	0.310
AST, U/L	18 (15–20.5)	19 (15–22.5)	18 (15.5–20)	0.637
TC, mmol/L	4.69 (4.09–5.36)	4.92 (4.4–5.28)	4.58 (3.94–5.49)	0.444
HDL, mmol/L	1.67 (1.4–1.92)	1.7 (1.37–1.99)	1.64 (1.42–1.89)	0.941
LDL, mmol/L	2.73 (2.27–3.38)	2.8 (2.36–3.24)	2.73 (2.18–3.5)	0.937
TG, mmol/L	0.97 (0.71–1.25)	1.07 (0.84–1.61)	0.91 (0.65–1.19)	0.057
eGFR, mL·min^{-1}·1.73m^{-2}	109 (9–118)	105 (94–119)	109 (101–115)	0.429
IL-29, pg/mL	12 (11–13)	12 (11–13)	12 (12–13)	0.427

* P value for comparison groups in accordance to microangiopathy occurrence ** χ^2 test; *** Yates corrected χ^2 test; Mann–Whitney U test in every other case, where it is not marked with **. Abbreviations: IL-29, interleukin 29 concentration; other see Table 2. A P value less than 0.05 was considered statistically significant. The bolded *p*-values are those which are statistically significant.

Supplementary Table 3. Correlations between various parameters and EGF, GDF-15, MMP-2 and IL-29 concentrations (Spearman's rank correlation analysis).

**Parameter**	**EGF**		**GDF-15**		**MMP-2**		**IL-29**	
	**r**	***P* value**	**r**	***P* value**	**r**	***P* value**	**r**	***P* value**
Age	0.09	0.360	0.09	0.457	-0.11	0.388	0.07	0.532
T1DM duration	0.07	0.478	-0.07	0.560	0.11	0.349	-0.05	0.691
SBP	0.21	0.035	0.03	0.782	0.09	0.477	-0.03	0.814
DBP	0.14	0.158	0.11	0.367	0.20	0.107	-0.03	0.786
BMI	0.11	0.267	-0.04	0.747	-0.05	0.657	0.08	0.498
HbA_{1c}	-0.02	0.857	0.14	0.246	-0.07	0.550	-0.06	0.614
hsCRP	0.14	0.177	0.27	0.027	0.21	0.090	0.07	0.545
TC	-0.11	0.263	-0.11	0.383	0.008	0.948	-0.23	0.041
HDL	-0.20	0.046	-0.12	0.328	0.25	0.035	-0.23	0.040
LDL	-0.05	0.588	-0.08	0.523	-0.12	0.322	-0.14	0.218
TG	0.02	0.876	0.15	0.215	-0.29	0.016	-0.13	0.244
eGFR	-0.11	0.255	-0.28	0.022	-0.22	0.064	-0.14	0.217
EGF	-	-	0.33	0.007	-0.01	0.958	0.05	0.655
GDF-15	0.33	0.007	-	-	0.27	0.020	0.06	0.679
MMP-2	-0.01	0.909	0.27	0.029	-	-	0.17	0.204
IL-29	0.05	0.655	0.06	0.679	0.17	0.204	-	-

Abbreviations: ALT, alanine transaminase; AST, aspartate transaminase; BMI, body mass index; DBP, diastolic blood pressure; EGF, epidermal growth factor concentration; eGFR, estimated glomerular filtration rate; GDF-15, growth/differentiation factor 15; HbA_{1c}, glycated hemoglobin; HDL, high-density lipoprotein; hsCRP, high sensitive C-reactive protein; IL-29, interleukin 29 concentration; IQR, interquartile range; LDL, low-density lipoprotein; Log_{10}EGF, decadic logarithm of epidermal growth factor concentration; MMP-2, metalloproteinase 2 concentration; SBP, systolic blood pressure; T1DM, type 1 diabetes; TC, total cholesterol; TG, triglycerides. A *p*-value less than 0.05 was considered statistically significant. The bolded *p*-values are those which are statistically significant.

Supplementary Table 4. Markers of diabetic kidney disease occurrence in multivariate logistic regression analysis with diabetic kidney disease as dependent variable and GDF-15, T1DM duration, HbA_{1c}, smoking, and BMI as independent variables. For the entire model $p = 0.0002$.

**Predictors**	**Odds ratio (95% confidence interwal)**	***P* value**
GDF-15	1.07 (1.01–1.12)	**0.015**
T1DM duration	1.63 (1.14–2.32)	**0.006**
HbA_{1c}	1.94 (0.54–7.01)	0.301
Smoking	4.16 (0.12–141.73)	0.420
BMI	0.81 (0.52–1.26)	0.342

Abbreviations: see Table 2. The bolded *p*-values are those which are statistically significant.
